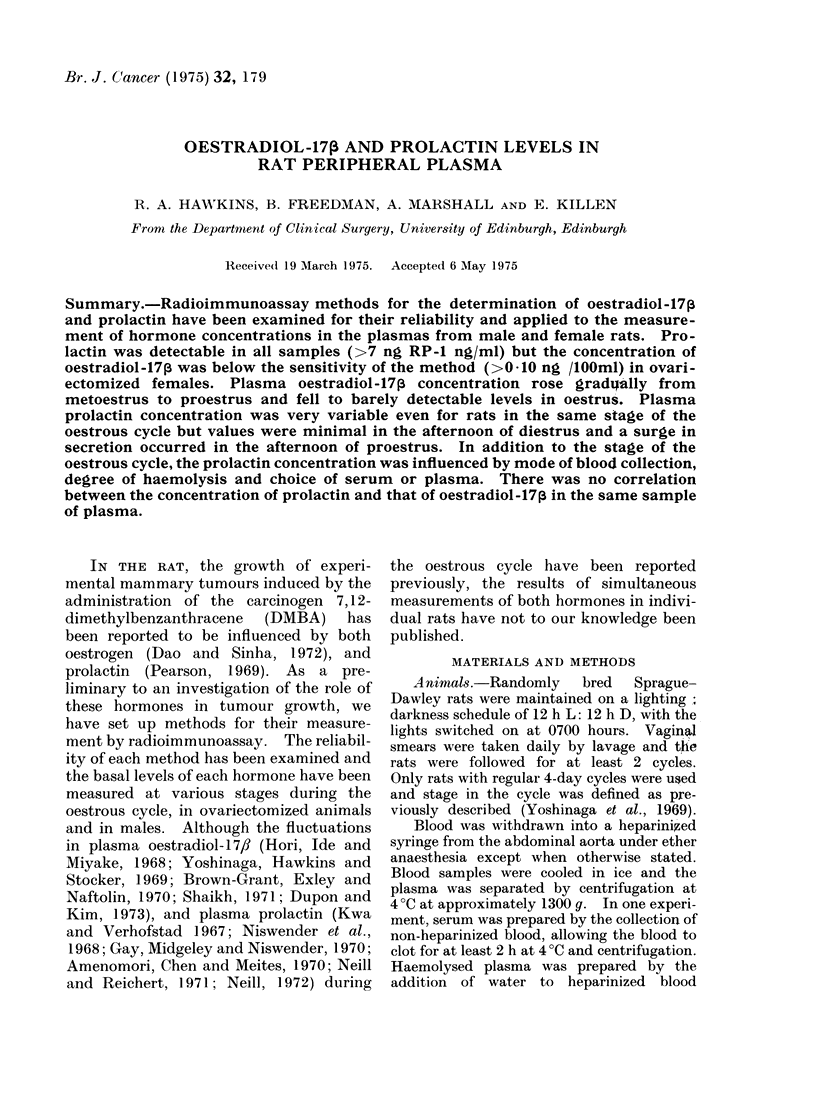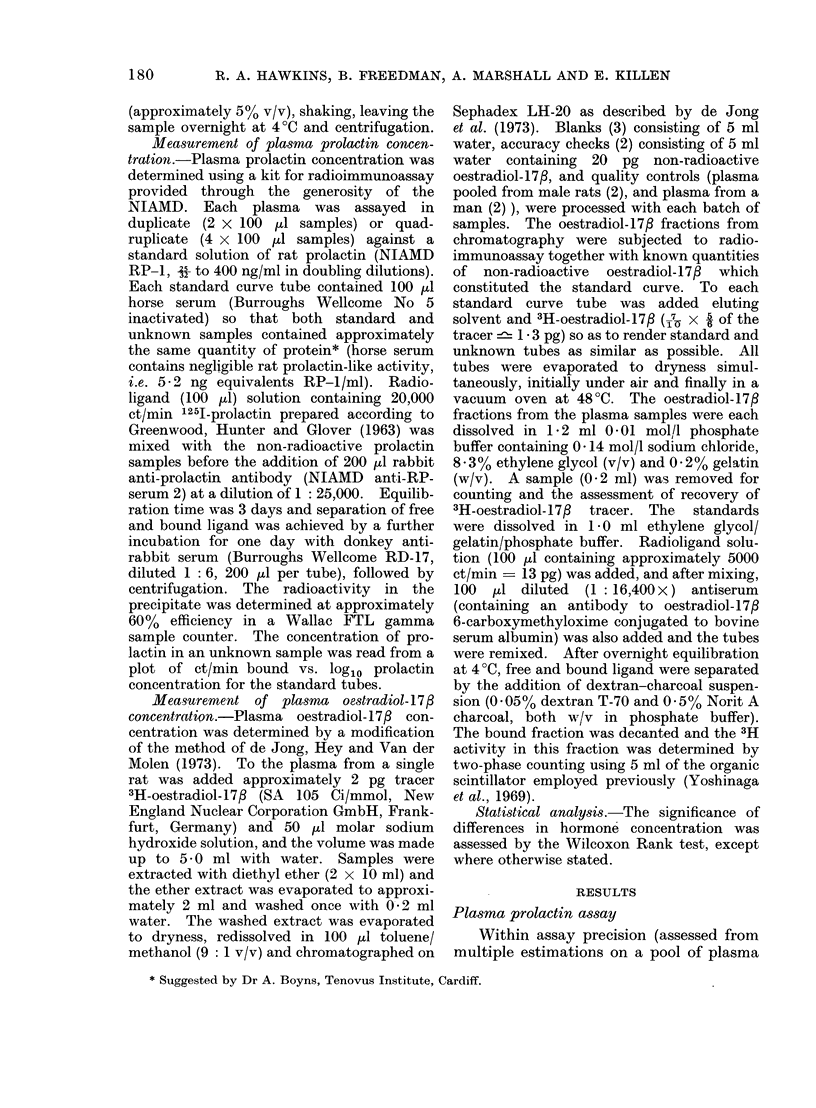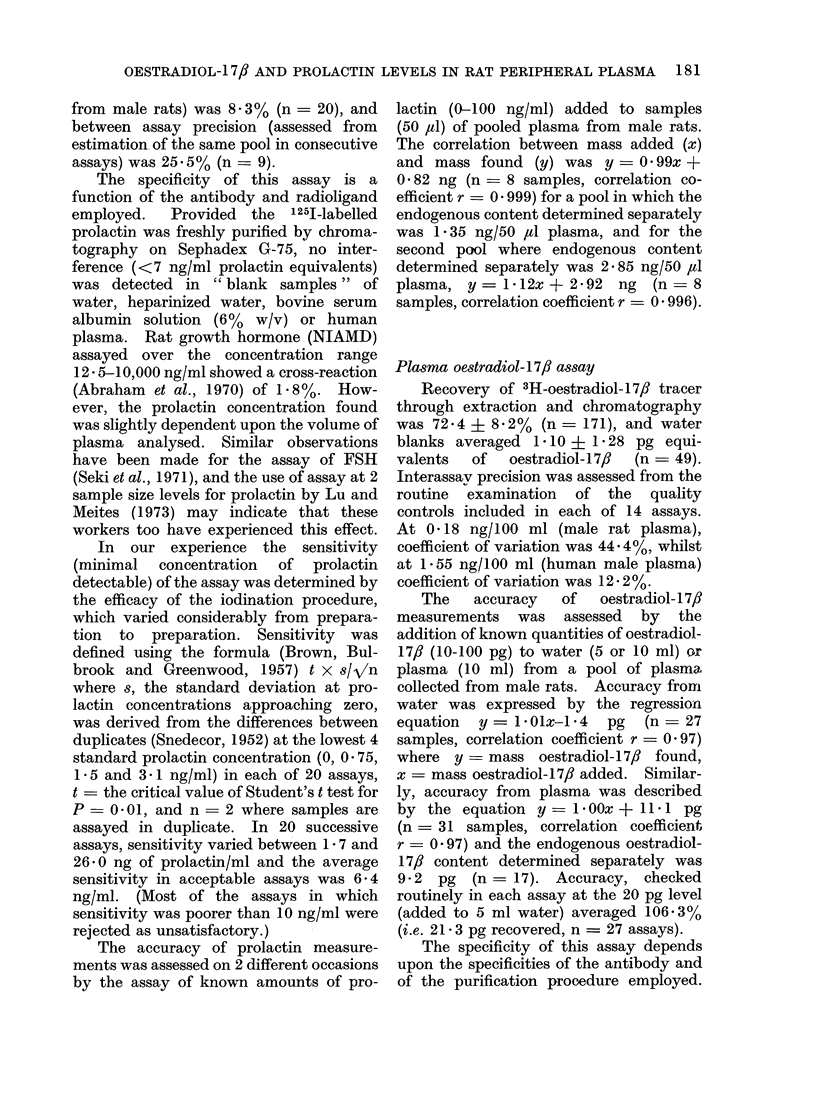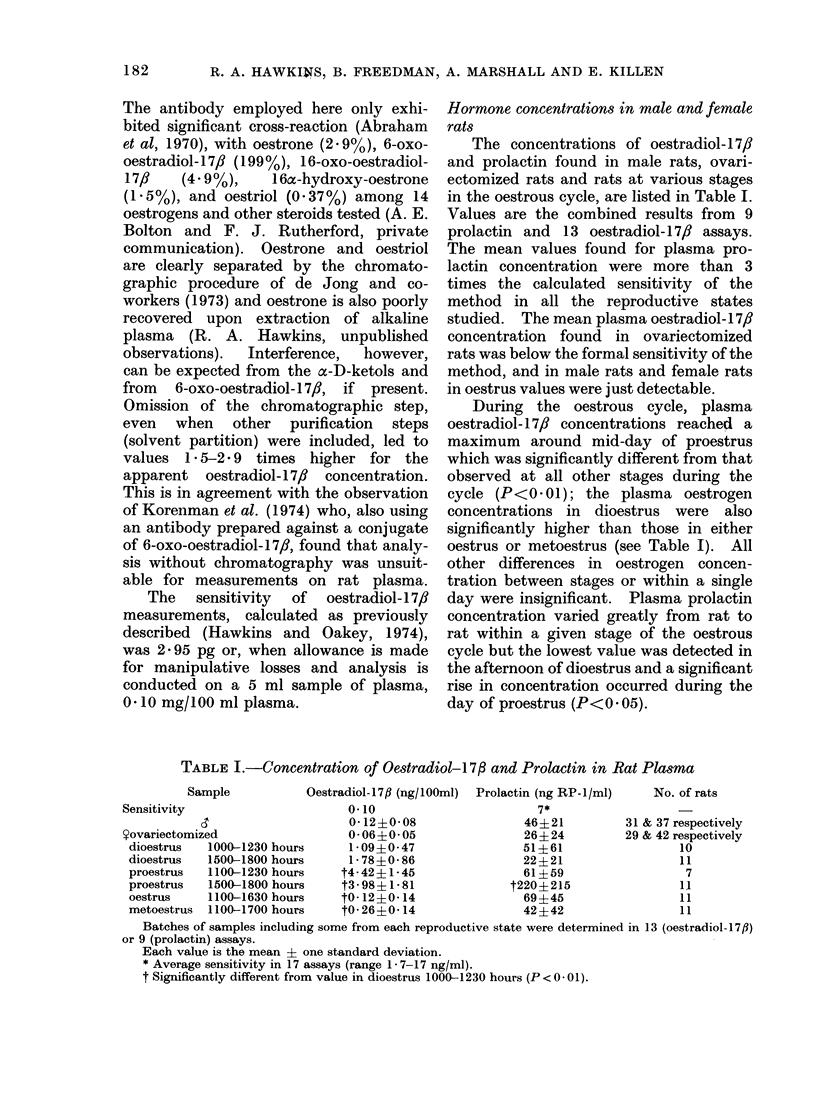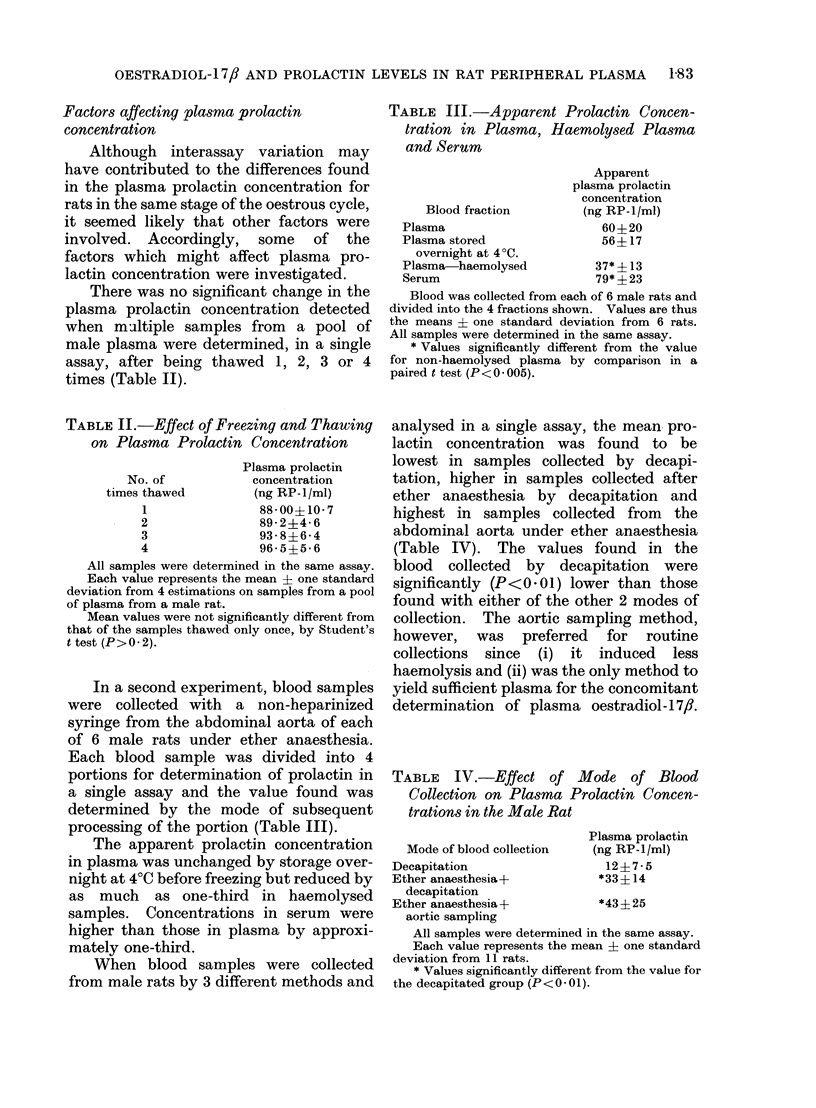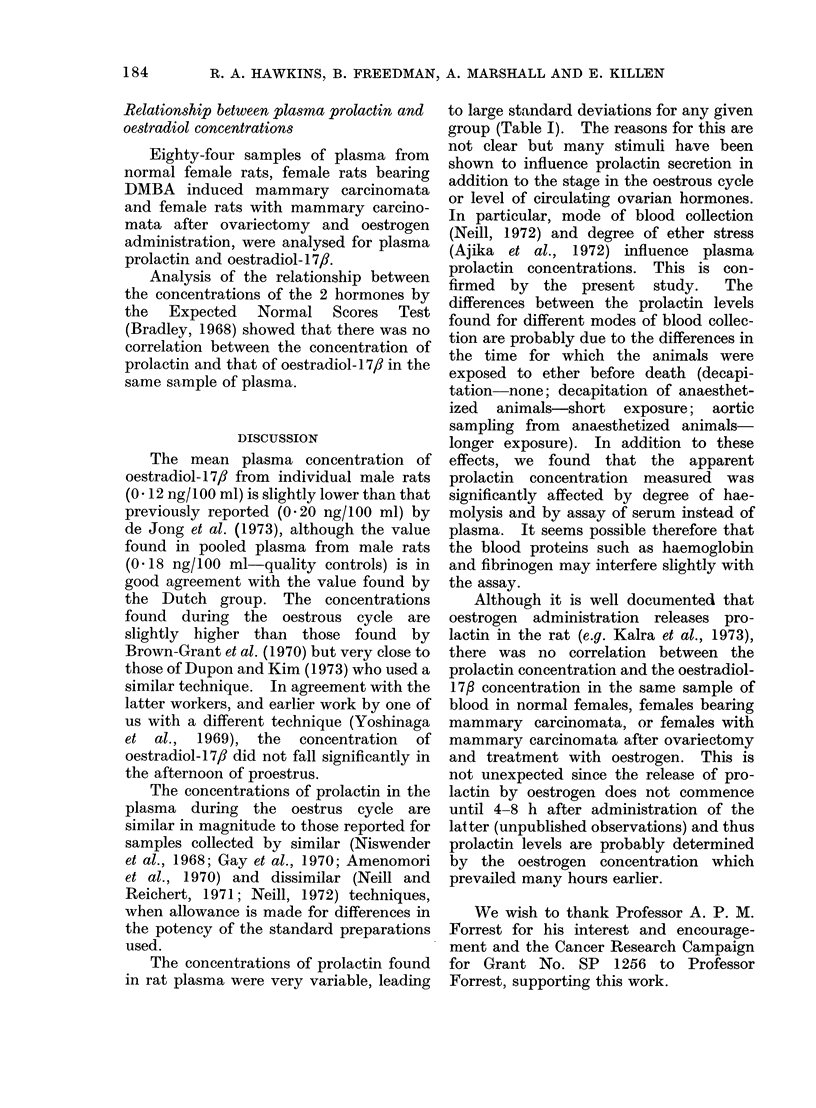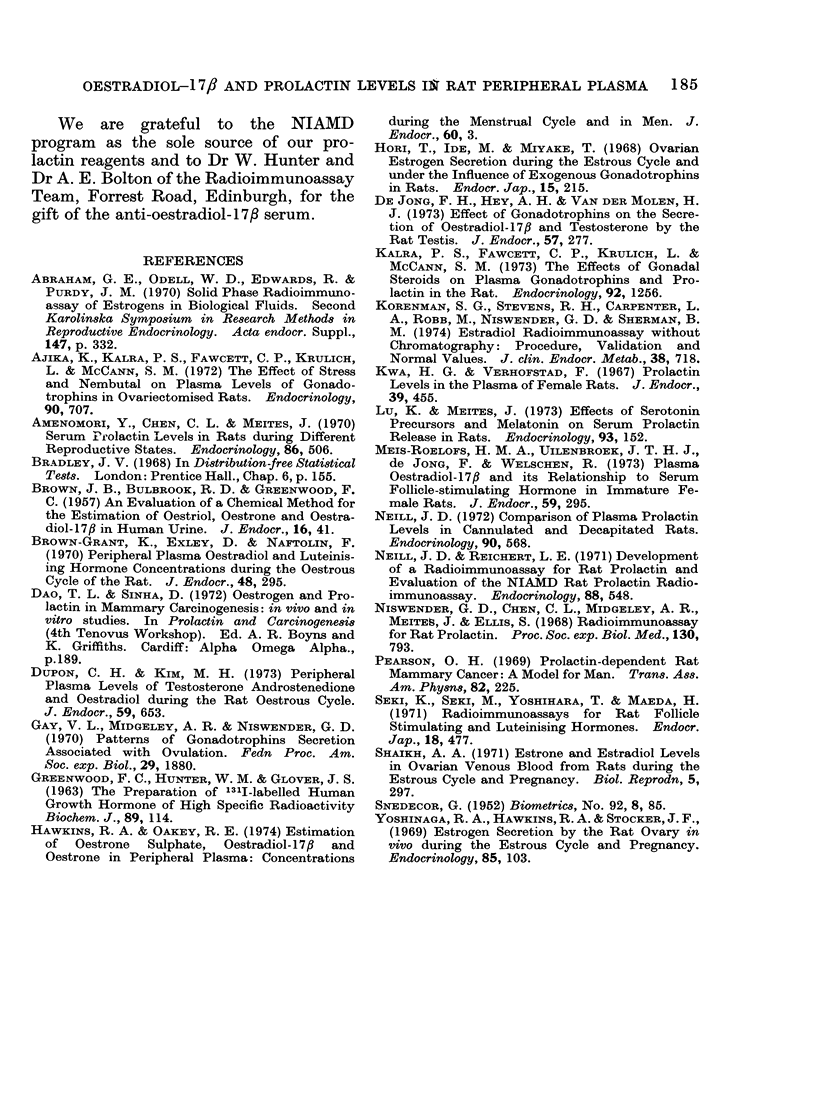# Oestradiol-17 beta and prolactin levels in rat peripheral plasma.

**DOI:** 10.1038/bjc.1975.147

**Published:** 1975-08

**Authors:** R. A. Hawkins, B. Freedman, A. Marshall, E. Killen

## Abstract

Radioimmunoassay methods for the determination of oestradiol-17 beta and prolactin have been examined for their reliability and applied to the measurement of hormone concentrations in the plasmas from male and female rats. Prolactin was detectable in all samples (greater than 7 ng RP-1 ng/ml) but the concentration of oestradiol-17 beta was below the sensitivity of the method (greater than 0-10 ng/100 ml) in ovariectomized females. Plasma oestradiol-17 beta concentration rose gradually from metoestrus to proestrus and fell to barely detectable levels in oestrus. Plasma prolactin concentration was very variable even for rats in the same stage of the oestrous cycle but values were minimal in the afternoon of diestrus and a surge in secretion occurred in the afternoon of proestrus. In addition to the stage of the oestrous cycle, the prolactin concentration was influenced by mode of blood collection, degree of haemolysis and choice of serum or plasma. There was no correlation between the concentration of prolactin and that of oestradiol-17 beta in the same sample of plasma.


*Br. J. Cancer* (1975) **32**, 179

### OESTRADIOL-17β AND PROLACTIN LEVELS IN RAT PERIPHERAL PLASMA

R. A. HAWKINS, B. FREEDMAN, A. MARSHALL AND E. KILLEN

*From the Department of Clinical Surgery, University of Edinburgh, Edinburgh*

Received 19 March 1975. Accepted 6 May 1975

**Summary.—Radioimmunoassay methods for the determination of oestradiol-17β and prolactin have been examined for their reliability and applied to the measurement of hormone concentrations in the plasmas from male and female rats. Prolactin was detectable in all samples (>7 ng RP-1 ng/ml) but the concentration of oestradiol-17β was below the sensitivity of the method (>0·10 ng /100ml) in ovariectomized females. Plasma oestradiol-17β concentration rose gradually from metoestrus to proestrus and fell to barely detectable levels in oestrus. Plasma prolactin concentration was very variable even for rats in the same stage of the oestrous cycle but values were minimal in the afternoon of diestrus and a surge in secretion occurred in the afternoon of proestrus. In addition to the stage of the oestrous cycle, the prolactin concentration was influenced by mode of blood collection, degree of haemolysis and choice of serum or plasma. There was no correlation between the concentration of prolactin and that of oestradiol-17β in the same sample of plasma.**

IN THE RAT, the growth of experimental mammary tumours induced by the administration of the carcinogen 7,12-dimethylbenzanthracene (DMBA) has been reported to be influenced by both oestrogen (Dao and Sinha, 1972), and prolactin (Pearson, 1969). As a preliminary to an investigation of the role of these hormones in tumour growth, we have set up methods for their measurement by radioimmunoassay. The reliability of each method has been examined and the basal levels of each hormone have been measured at various stages during the oestrous cycle, in ovariectomized animals and in males. Although the fluctuations in plasma oestradiol-17*β* (Hori, Ide and Miyake, 1968; Yoshinaga, Hawkins and Stocker, 1969; Brown-Grant, Exley and Naftolin, 1970; Shaikh, 1971; Dupon and Kim, 1973), and plasma prolactin (Kwa and Verhofstad 1967; Niswender *et al.*, 1968; Gay, Midgeley and Niswender, 1970; Amenomori, Chen and Meites, 1970; Neill and Reichert, 1971; Neill, 1972) during the oestrous cycle have been reported previously, the results of simultaneous measurements of both hormones in individual rats have not to our knowledge been published.

### MATERIALS AND METHODS

*Animals.*—Randomly bred Sprague–Dawley rats were maintained on a lighting : darkness schedule of 12 h L: 12 h D, with the lights switched on at 0700 hours. Vaginal smears were taken daily by lavage and the rats were followed for at least 2 cycles. Only rats with regular 4-day cycles were used and stage in the cycle was defined as previously described (Yoshinaga *et al.*, 1969).

Blood was withdrawn into a heparinized syringe from the abdominal aorta under ether anaesthesia except when otherwise stated. Blood samples were cooled in ice and the plasma was separated by centrifugation at 4 °C at approximately 1300 *g*. In one experiment, serum was prepared by the collection of non-heparinized blood, allowing the blood to clot for at least 2 h at 4 °C and centrifugation. Haemolysed plasma was prepared by the addition of water to heparinized blood

(approximately 5% v/v), shaking, leaving the sample overnight at 4 °C and centrifugation.

*Measurement of plasma prolactin concentration.*—Plasma prolactin concentration was determined using a kit for radioimmunoassay provided through the generosity of the NIAMD. Each plasma was assayed in duplicate (2 × 100 μl samples) or quadruplicate (4 × 100 μl samples) against a standard solution of rat prolactin (NIAMD RP–1, $\frac{25}{32}$ to 400 ng/ml in doubling dilutions). Each standard curve tube contained 100 μl horse serum (Burroughs Wellcome No 5 inactivated) so that both standard and unknown samples contained approximately the same quantity of protein* (horse serum contains negligible rat prolactin-like activity, *i.e.* 5·2 ng equivalents RP–1/ml). Radioligand (100 μl) solution containing 20,000 ct/min $^{125}$I-prolactin prepared according to Greenwood, Hunter and Glover (1963) was mixed with the non-radioactive prolactin samples before the addition of 200 μl rabbit anti-prolactin antibody (NIAMD anti-RP-serum 2) at a dilution of 1 : 25,000. Equilibration time was 3 days and separation of free and bound ligand was achieved by a further incubation for one day with donkey anti-rabbit serum (Burroughs Wellcome RD-17, diluted 1 : 6, 200 μl per tube), followed by centrifugation. The radioactivity in the precipitate was determined at approximately 60% efficiency in a Wallac FTL gamma sample counter. The concentration of prolactin in an unknown sample was read from a plot of ct/min bound vs. $\log_{10}$ prolactin concentration for the standard tubes.

*Measurement of plasma oestradiol-17β concentration.*—Plasma oestradiol-17β concentration was determined by a modification of the method of de Jong, Hey and Van der Molen (1973). To the plasma from a single rat was added approximately 2 pg tracer $^3$H-oestradiol-17β (SA 105 Ci/mmol, New England Nuclear Corporation GmbH, Frankfurt, Germany) and 50 μl molar sodium hydroxide solution, and the volume was made up to 5·0 ml with water. Samples were extracted with diethyl ether (2 × 10 ml) and the ether extract was evaporated to approximately 2 ml and washed once with 0·2 ml water. The washed extract was evaporated to dryness, redissolved in 100 μl toluene/methanol (9 : 1 v/v) and chromatographed on Sephadex LH-20 as described by de Jong *et al.* (1973). Blanks (3) consisting of 5 ml water, accuracy checks (2) consisting of 5 ml water containing 20 pg non-radioactive oestradiol-17β, and quality controls (plasma pooled from male rats (2), and plasma from a man (2) ), were processed with each batch of samples. The oestradiol-17β fractions from chromatography were subjected to radioimmunoassay together with known quantities of non-radioactive oestradiol-17β which constituted the standard curve. To each standard curve tube was added eluting solvent and $^3$H-oestradiol-17β ($\frac{7}{10} \times \frac{5}{6}$ of the tracer ≏ 1·3 pg) so as to render standard and unknown tubes as similar as possible. All tubes were evaporated to dryness simultaneously, initially under air and finally in a vacuum oven at 48 °C. The oestradiol-17β fractions from the plasma samples were each dissolved in 1·2 ml 0·01 mol/l phosphate buffer containing 0·14 mol/l sodium chloride, 8·3% ethylene glycol (v/v) and 0·2% gelatin (w/v). A sample (0·2 ml) was removed for counting and the assessment of recovery of $^3$H-oestradiol-17β tracer. The standards were dissolved in 1·0 ml ethylene glycol/gelatin/phosphate buffer. Radioligand solution (100 μl containing approximately 5000 ct/min = 13 pg) was added, and after mixing, 100 μl diluted (1 : 16,400×) antiserum (containing an antibody to oestradiol-17β 6-carboxymethyloxime conjugated to bovine serum albumin) was also added and the tubes were remixed. After overnight equilibration at 4 °C, free and bound ligand were separated by the addition of dextran–charcoal suspension (0·05% dextran T-70 and 0·5% Norit A charcoal, both w/v in phosphate buffer). The bound fraction was decanted and the $^3$H activity in this fraction was determined by two-phase counting using 5 ml of the organic scintillator employed previously (Yoshinaga *et al.*, 1969).

*Statistical analysis.*—The significance of differences in hormone concentration was assessed by the Wilcoxon Rank test, except where otherwise stated.

### RESULTS

### *Plasma prolactin assay*

Within assay precision (assessed from multiple estimations on a pool of plasma

* Suggested by Dr A. Boyns, Tenovus Institute, Cardiff.

from male rats) was 8·3% (n = 20), and between assay precision (assessed from estimation of the same pool in consecutive assays) was 25·5% (n = 9).

The specificity of this assay is a function of the antibody and radioligand employed. Provided the $^{125}$I-labelled prolactin was freshly purified by chromatography on Sephadex G-75, no interference (<7 ng/ml prolactin equivalents) was detected in "blank samples" of water, heparinized water, bovine serum albumin solution (6% w/v) or human plasma. Rat growth hormone (NIAMD) assayed over the concentration range 12·5–10,000 ng/ml showed a cross-reaction (Abraham *et al.*, 1970) of 1·8%. However, the prolactin concentration found was slightly dependent upon the volume of plasma analysed. Similar observations have been made for the assay of FSH (Seki *et al.*, 1971), and the use of assay at 2 sample size levels for prolactin by Lu and Meites (1973) may indicate that these workers too have experienced this effect.

In our experience the sensitivity (minimal concentration of prolactin detectable) of the assay was determined by the efficacy of the iodination procedure, which varied considerably from preparation to preparation. Sensitivity was defined using the formula (Brown, Bulbrook and Greenwood, 1957) $t \times s/\sqrt{n}$ where $s$, the standard deviation at prolactin concentrations approaching zero, was derived from the differences between duplicates (Snedecor, 1952) at the lowest 4 standard prolactin concentration (0, 0·75, 1·5 and 3·1 ng/ml) in each of 20 assays, $t$ = the critical value of Student's $t$ test for $P = 0{\cdot}01$, and n = 2 where samples are assayed in duplicate. In 20 successive assays, sensitivity varied between 1·7 and 26·0 ng of prolactin/ml and the average sensitivity in acceptable assays was 6·4 ng/ml. (Most of the assays in which sensitivity was poorer than 10 ng/ml were rejected as unsatisfactory.)

The accuracy of prolactin measurements was assessed on 2 different occasions by the assay of known amounts of prolactin (0–100 ng/ml) added to samples (50 $\mu$l) of pooled plasma from male rats. The correlation between mass added ($x$) and mass found ($y$) was $y = 0{\cdot}99x + 0{\cdot}82$ ng (n = 8 samples, correlation coefficient $r = 0{\cdot}999$) for a pool in which the endogenous content determined separately was 1·35 ng/50 $\mu$l plasma, and for the second pool where endogenous content determined separately was 2·85 ng/50 $\mu$l plasma, $y = 1{\cdot}12x + 2{\cdot}92$ ng (n = 8 samples, correlation coefficient $r = 0{\cdot}996$).

*Plasma oestradiol*-17$\beta$ *assay*

Recovery of $^3$H-oestradiol-17$\beta$ tracer through extraction and chromatography was 72·4 ± 8·2% (n = 171), and water blanks averaged 1·10 ± 1·28 pg equivalents of oestradiol-17$\beta$ (n = 49). Interassay precision was assessed from the routine examination of the quality controls included in each of 14 assays. At 0·18 ng/100 ml (male rat plasma), coefficient of variation was 44·4%, whilst at 1·55 ng/100 ml (human male plasma) coefficient of variation was 12·2%.

The accuracy of oestradiol-17$\beta$ measurements was assessed by the addition of known quantities of oestradiol-17$\beta$ (10-100 pg) to water (5 or 10 ml) or plasma (10 ml) from a pool of plasma collected from male rats. Accuracy from water was expressed by the regression equation $y = 1{\cdot}01x - 1{\cdot}4$ pg (n = 27 samples, correlation coefficient $r = 0{\cdot}97$) where $y$ = mass oestradiol-17$\beta$ found, $x$ = mass oestradiol-17$\beta$ added. Similarly, accuracy from plasma was described by the equation $y = 1{\cdot}00x + 11{\cdot}1$ pg (n = 31 samples, correlation coefficient $r = 0{\cdot}97$) and the endogenous oestradiol-17$\beta$ content determined separately was 9·2 pg (n = 17). Accuracy, checked routinely in each assay at the 20 pg level (added to 5 ml water) averaged 106·3% (*i.e.* 21·3 pg recovered, n = 27 assays).

The specificity of this assay depends upon the specificities of the antibody and of the purification procedure employed.

The antibody employed here only exhibited significant cross-reaction (Abraham *et al*, 1970), with oestrone (2·9%), 6-oxo-oestradiol-17β (199%), 16-oxo-oestradiol-17β (4·9%), 16α-hydroxy-oestrone (1·5%), and oestriol (0·37%) among 14 oestrogens and other steroids tested (A. E. Bolton and F. J. Rutherford, private communication). Oestrone and oestriol are clearly separated by the chromatographic procedure of de Jong and co-workers (1973) and oestrone is also poorly recovered upon extraction of alkaline plasma (R. A. Hawkins, unpublished observations). Interference, however, can be expected from the α-D-ketols and from 6-oxo-oestradiol-17β, if present. Omission of the chromatographic step, even when other purification steps (solvent partition) were included, led to values 1·5–2·9 times higher for the apparent oestradiol-17β concentration. This is in agreement with the observation of Korenman *et al.* (1974) who, also using an antibody prepared against a conjugate of 6-oxo-oestradiol-17β, found that analysis without chromatography was unsuitable for measurements on rat plasma.

The sensitivity of oestradiol-17β measurements, calculated as previously described (Hawkins and Oakey, 1974), was 2·95 pg or, when allowance is made for manipulative losses and analysis is conducted on a 5 ml sample of plasma, 0·10 mg/100 ml plasma.

*Hormone concentrations in male and female rats*

The concentrations of oestradiol-17β and prolactin found in male rats, ovariectomized rats and rats at various stages in the oestrous cycle, are listed in Table I. Values are the combined results from 9 prolactin and 13 oestradiol-17β assays. The mean values found for plasma prolactin concentration were more than 3 times the calculated sensitivity of the method in all the reproductive states studied. The mean plasma oestradiol-17β concentration found in ovariectomized rats was below the formal sensitivity of the method, and in male rats and female rats in oestrus values were just detectable.

During the oestrous cycle, plasma oestradiol-17β concentrations reached a maximum around mid-day of proestrus which was significantly different from that observed at all other stages during the cycle ($P<0·01$); the plasma oestrogen concentrations in dioestrus were also significantly higher than those in either oestrus or metoestrus (see Table I). All other differences in oestrogen concentration between stages or within a single day were insignificant. Plasma prolactin concentration varied greatly from rat to rat within a given stage of the oestrous cycle but the lowest value was detected in the afternoon of dioestrus and a significant rise in concentration occurred during the day of proestrus ($P<0·05$).

TABLE I.—*Concentration of Oestradiol–*17β *and Prolactin in Rat Plasma*

| Sample | | Oestradiol-17β (ng/100ml) | Prolactin (ng RP-1/ml) | No. of rats |
|---|---|---|---|---|
| Sensitivity | | 0·10 | 7* | — |
| ♂ | | 0·12±0·08 | 46±21 | 31 & 37 respectively |
| ♀ovariectomized | | 0·06±0·05 | 26±24 | 29 & 42 respectively |
| dioestrus | 1000–1230 hours | 1·09±0·47 | 51±61 | 10 |
| dioestrus | 1500–1800 hours | 1·78±0·86 | 22±21 | 11 |
| proestrus | 1100–1230 hours | †4·42±1·45 | 61±59 | 7 |
| proestrus | 1500–1800 hours | †3·98±1·81 | †220±215 | 11 |
| oestrus | 1100–1630 hours | †0·12±0·14 | 69±45 | 11 |
| metoestrus | 1100–1700 hours | †0·26±0·14 | 42±42 | 11 |

Batches of samples including some from each reproductive state were determined in 13 (oestradiol-17β) or 9 (prolactin) assays.

Each value is the mean ± one standard deviation.

* Average sensitivity in 17 assays (range 1·7–17 ng/ml).

† Significantly different from value in dioestrus 1000–1230 hours ($P<0·01$).

*Factors affecting plasma prolactin concentration*

Although interassay variation may have contributed to the differences found in the plasma prolactin concentration for rats in the same stage of the oestrous cycle, it seemed likely that other factors were involved. Accordingly, some of the factors which might affect plasma prolactin concentration were investigated.

There was no significant change in the plasma prolactin concentration detected when multiple samples from a pool of male plasma were determined, in a single assay, after being thawed 1, 2, 3 or 4 times (Table II).

TABLE II.—*Effect of Freezing and Thawing on Plasma Prolactin Concentration*

| No. of times thawed | Plasma prolactin concentration (ng RP-1/ml) |
|---|---|
| 1 | 88·00±10·7 |
| 2 | 89·2±4·6 |
| 3 | 93·8±6·4 |
| 4 | 96·5±5·6 |

All samples were determined in the same assay.

Each value represents the mean ± one standard deviation from 4 estimations on samples from a pool of plasma from a male rat.

Mean values were not significantly different from that of the samples thawed only once, by Student's $t$ test ($P>0\cdot2$).

In a second experiment, blood samples were collected with a non-heparinized syringe from the abdominal aorta of each of 6 male rats under ether anaesthesia. Each blood sample was divided into 4 portions for determination of prolactin in a single assay and the value found was determined by the mode of subsequent processing of the portion (Table III).

The apparent prolactin concentration in plasma was unchanged by storage overnight at 4°C before freezing but reduced by as much as one-third in haemolysed samples. Concentrations in serum were higher than those in plasma by approximately one-third.

When blood samples were collected from male rats by 3 different methods and analysed in a single assay, the mean prolactin concentration was found to be lowest in samples collected by decapitation, higher in samples collected after ether anaesthesia by decapitation and highest in samples collected from the abdominal aorta under ether anaesthesia (Table IV). The values found in the blood collected by decapitation were significantly ($P<0\cdot01$) lower than those found with either of the other 2 modes of collection. The aortic sampling method, however, was preferred for routine collections since (i) it induced less haemolysis and (ii) was the only method to yield sufficient plasma for the concomitant determination of plasma oestradiol-17$\beta$.

TABLE III.—*Apparent Prolactin Concentration in Plasma, Haemolysed Plasma and Serum*

| Blood fraction | Apparent plasma prolactin concentration (ng RP-1/ml) |
|---|---|
| Plasma | 60±20 |
| Plasma stored overnight at 4°C. | 56±17 |
| Plasma—haemolysed | 37*±13 |
| Serum | 79*±23 |

Blood was collected from each of 6 male rats and divided into the 4 fractions shown. Values are thus the means ± one standard deviation from 6 rats. All samples were determined in the same assay.

* Values significantly different from the value for non-haemolysed plasma by comparison in a paired $t$ test ($P<0\cdot005$).

TABLE IV.—*Effect of Mode of Blood Collection on Plasma Prolactin Concentrations in the Male Rat*

| Mode of blood collection | Plasma prolactin (ng RP-1/ml) |
|---|---|
| Decapitation | 12±7·5 |
| Ether anaesthesia + decapitation | *33±14 |
| Ether anaesthesia + aortic sampling | *43±25 |

All samples were determined in the same assay.

Each value represents the mean ± one standard deviation from 11 rats.

* Values significantly different from the value for the decapitated group ($P<0\cdot01$).

*Relationship between plasma prolactin and oestradiol concentrations*

Eighty-four samples of plasma from normal female rats, female rats bearing DMBA induced mammary carcinomata and female rats with mammary carcinomata after ovariectomy and oestrogen administration, were analysed for plasma prolactin and oestradiol-17$\beta$.

Analysis of the relationship between the concentrations of the 2 hormones by the Expected Normal Scores Test (Bradley, 1968) showed that there was no correlation between the concentration of prolactin and that of oestradiol-17$\beta$ in the same sample of plasma.

### DISCUSSION

The mean plasma concentration of oestradiol-17$\beta$ from individual male rats (0·12 ng/100 ml) is slightly lower than that previously reported (0·20 ng/100 ml) by de Jong *et al.* (1973), although the value found in pooled plasma from male rats (0·18 ng/100 ml—quality controls) is in good agreement with the value found by the Dutch group. The concentrations found during the oestrous cycle are slightly higher than those found by Brown-Grant *et al.* (1970) but very close to those of Dupon and Kim (1973) who used a similar technique. In agreement with the latter workers, and earlier work by one of us with a different technique (Yoshinaga *et al.*, 1969), the concentration of oestradiol-17$\beta$ did not fall significantly in the afternoon of proestrus.

The concentrations of prolactin in the plasma during the oestrus cycle are similar in magnitude to those reported for samples collected by similar (Niswender *et al.*, 1968; Gay *et al.*, 1970; Amenomori *et al.*, 1970) and dissimilar (Neill and Reichert, 1971; Neill, 1972) techniques, when allowance is made for differences in the potency of the standard preparations used.

The concentrations of prolactin found in rat plasma were very variable, leading to large standard deviations for any given group (Table I). The reasons for this are not clear but many stimuli have been shown to influence prolactin secretion in addition to the stage in the oestrous cycle or level of circulating ovarian hormones. In particular, mode of blood collection (Neill, 1972) and degree of ether stress (Ajika *et al.*, 1972) influence plasma prolactin concentrations. This is confirmed by the present study. The differences between the prolactin levels found for different modes of blood collection are probably due to the differences in the time for which the animals were exposed to ether before death (decapitation—none; decapitation of anaesthetized animals—short exposure; aortic sampling from anaesthetized animals—longer exposure). In addition to these effects, we found that the apparent prolactin concentration measured was significantly affected by degree of haemolysis and by assay of serum instead of plasma. It seems possible therefore that the blood proteins such as haemoglobin and fibrinogen may interfere slightly with the assay.

Although it is well documented that oestrogen administration releases prolactin in the rat (*e.g.* Kalra *et al.*, 1973), there was no correlation between the prolactin concentration and the oestradiol-17$\beta$ concentration in the same sample of blood in normal females, females bearing mammary carcinomata, or females with mammary carcinomata after ovariectomy and treatment with oestrogen. This is not unexpected since the release of prolactin by oestrogen does not commence until 4–8 h after administration of the latter (unpublished observations) and thus prolactin levels are probably determined by the oestrogen concentration which prevailed many hours earlier.

We wish to thank Professor A. P. M. Forrest for his interest and encouragement and the Cancer Research Campaign for Grant No. SP 1256 to Professor Forrest, supporting this work.

We are grateful to the NIAMD program as the sole source of our prolactin reagents and to Dr W. Hunter and Dr A. E. Bolton of the Radioimmunoassay Team, Forrest Road, Edinburgh, for the gift of the anti-oestradiol-17β serum.

### REFERENCES

ABRAHAM, G. E., ODELL, W. D., EDWARDS, R. & PURDY, J. M. (1970) Solid Phase Radioimmunoassay of Estrogens in Biological Fluids. Second *Karolinska Symposium in Research Methods in Reproductive Endocrinology. Acta endocr.* Suppl., **147**, p. 332.

AJIKA, K., KALRA, P. S., FAWCETT, C. P., KRULICH, L. & MCCANN, S. M. (1972) The Effect of Stress and Nembutal on Plasma Levels of Gonadotrophins in Ovariectomised Rats. *Endocrinology*, **90**, 707.

AMENOMORI, Y., CHEN, C. L. & MEITES, J. (1970) Serum Prolactin Levels in Rats during Different Reproductive States. *Endocrinology*, **86**, 506.

BRADLEY, J. V. (1968) In *Distribution-free Statistical Tests.* London: Prentice Hall., Chap. 6, p. 155.

BROWN, J. B., BULBROOK, R. D. & GREENWOOD, F. C. (1957) An Evaluation of a Chemical Method for the Estimation of Oestriol, Oestrone and Oestradiol-17β in Human Urine. *J. Endocr.*, **16**, 41.

BROWN-GRANT, K., EXLEY, D. & NAFTOLIN, F. (1970) Peripheral Plasma Oestradiol and Luteinising Hormone Concentrations during the Oestrous Cycle of the Rat. *J. Endocr.*, **48**, 295.

DAO, T. L. & SINHA, D. (1972) Oestrogen and Prolactin in Mammary Carcinogenesis: *in vivo* and *in vitro* studies. In *Prolactin and Carcinogenesis* (4th Tenovus Workshop). Ed. A. R. Boyns and K. Griffiths. Cardiff: Alpha Omega Alpha., p.189.

DUPON, C. H. & KIM, M. H. (1973) Peripheral Plasma Levels of Testosterone Androstenedione and Oestradiol during the Rat Oestrous Cycle. *J. Endocr.*, **59**, 653.

GAY, V. L., MIDGELEY, A. R. & NISWENDER, G. D. (1970) Patterns of Gonadotrophins Secretion Associated with Ovulation. *Fedn Proc. Am. Soc. exp. Biol.*, **29**, 1880.

GREENWOOD, F. C., HUNTER, W. M. & GLOVER, J. S. (1963) The Preparation of $^{131}$I-labelled Human Growth Hormone of High Specific Radioactivity *Biochem. J.*, **89**, 114.

HAWKINS, R. A. & OAKEY, R. E. (1974) Estimation of Oestrone Sulphate, Oestradiol-17β and Oestrone in Peripheral Plasma: Concentrations during the Menstrual Cycle and in Men. *J. Endocr.*, **60**, 3.

HORI, T., IDE, M. & MIYAKE, T. (1968) Ovarian Estrogen Secretion during the Estrous Cycle and under the Influence of Exogenous Gonadotrophins in Rats. *Endocr. Jap.*, **15**, 215.

DE JONG, F. H., HEY, A. H. & VAN DER MOLEN, H. J. (1973) Effect of Gonadotrophins on the Secretion of Oestradiol-17β and Testosterone by the Rat Testis. *J. Endocr.*, **57**, 277.

KALRA, P. S., FAWCETT, C. P., KRULICH, L. & MCCANN, S. M. (1973) The Effects of Gonadal Steroids on Plasma Gonadotrophins and Prolactin in the Rat. *Endocrinology*, **92**, 1256.

KORENMAN, S. G., STEVENS, R. H., CARPENTER, L. A., ROBB, M., NISWENDER, G. D. & SHERMAN, B. M. (1974) Estradiol Radioimmunoassay without Chromatography: Procedure, Validation and Normal Values. *J. clin. Endocr. Metab.*, **38**, 718.

KWA, H. G. & VERHOFSTAD, F. (1967) Prolactin Levels in the Plasma of Female Rats. *J. Endocr.*, **39**, 455.

LU, K. & MEITES, J. (1973) Effects of Serotonin Precursors and Melatonin on Serum Prolactin Release in Rats. *Endocrinology*, **93**, 152.

MEIS-ROELOFS, H. M. A., UILENBROEK, J. T. H. J., de JONG, F. & WELSCHEN, R. (1973) Plasma Oestradiol-17β and its Relationship to Serum Follicle-stimulating Hormone in Immature Female Rats. *J. Endocr.*, **59**, 295.

NEILL, J. D. (1972) Comparison of Plasma Prolactin Levels in Cannulated and Decapitated Rats. *Endocrinology*, **90**, 568.

NEILL, J. D. & REICHERT, L. E. (1971) Development of a Radioimmunoassay for Rat Prolactin and Evaluation of the NIAMD Rat Prolactin Radioimmunoassay. *Endocrinology*, **88**, 548.

NISWENDER, G. D., CHEN, C. L., MIDGELEY, A. R., MEITES, J. & ELLIS, S. (1968) Radioimmunoassay for Rat Prolactin. *Proc. Soc. exp. Biol. Med.*, **130**, 793.

PEARSON, O. H. (1969) Prolactin-dependent Rat Mammary Cancer: A Model for Man. *Trans. Ass. Am. Physns*, **82**, 225.

SEKI, K., SEKI, M., YOSHIHARA, T. & MAEDA, H. (1971) Radioimmunoassays for Rat Follicle Stimulating and Luteinising Hormones. *Endocr. Jap.*, **18**, 477.

SHAIKH, A. A. (1971) Estrone and Estradiol Levels in Ovarian Venous Blood from Rats during the Estrous Cycle and Pregnancy. *Biol. Reprodn*, **5**, 297.

SNEDECOR, G. (1952) *Biometrics*, No. 92, **8**, 85.

YOSHINAGA, R. A., HAWKINS, R. A. & STOCKER, J. F., (1969) Estrogen Secretion by the Rat Ovary *in vivo* during the Estrous Cycle and Pregnancy. *Endocrinology*, **85**, 103.